# Demethylzeylasteral inhibits proliferation, migration, and invasion through FBXW7/c‐Myc axis in gastric cancer

**DOI:** 10.1002/mco2.73

**Published:** 2021-06-03

**Authors:** Yongsen Li, Yongyue Su, Yuzu Zhao, Xiaosong Hu, Gaichao Zhao, Jiang He, Sicheng Wan, Muhan Lü, Hongjuan Cui

**Affiliations:** ^1^ State Key Laboratory of Silkworm Genome Biology College of Sericulture Textile and Biomass sciences Southwest University Chongqing China; ^2^ Department of Orthopaedic 920th Hospital of Joint Logistics Support Force of Chinese People's Liberation Army Kunming China; ^3^ Department of Gastroenterology The Affiliated Hospital of Southwest Medical University Luzhou China; ^4^ Cancer Centre Medical Research Institute Southwest University Chongqing China

**Keywords:** chemosensitivity, c‐Myc, demethylzeylasteral, FBXW7, gastric cancer

## Abstract

Gastric cancer (GC) is one of the most familiar malignancy in the digestive system. Demethylzeylasteral (Dem), a natural functional monomer extracted from *Tripterygium wilfordii* Hook F, shows anti‐tumor effects in a variety of cancers, including GC, however, with the underlying mechanism poorly understood. In our study, we show that Dem inhibits the proliferation, migration, and invasion of GC cells, which are mediated by down‐regulating c‐Myc protein levels. Mechanistically, Dem reduces the stability of c‐Myc by up‐regulating FBXW7, an E3 ubiquitin ligase. Moreover, in xenograft tumor model experiment, Dem also inhibits GC, which depends on suppressing c‐Myc expression. Finally, Dem enhances GC cell chemosensitivity to the combination treatment of 5‐Fluorouracil (5‐Fu) and doxorubicin (DOX) in vitro. Together, Dem exerts anti‐neoplastic activities through destabilizing and suppressing c‐Myc, establishing a theory foundation for using it in future treatment of GC.

## Graphical abstract

Schematic diagram of mechanism of demethylzeylasteral (a natural functional monomer) treatment in gastric cancer. Demethylzeylasteral promotes the degradation of c‐Myc through up‐regulating the expression level of FBXW7 (an E3 ubiquitin ligase) and consequent ubiquitylization of c‐Myc, which in turn inhibits cell proliferation, migration and invasion, and therefore, depresses the growth of gastric cancer.

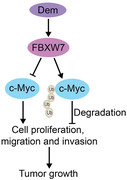

## 1 INTRODUCTION

Gastric cancer (GC) is a prevalent and heterogeneous tumor disease, ranking the fifth most diagnosed cancer and the fourth primary cause of cancer‐related death in the world.^1^, ^2^ The early clinical symptoms of GC are not obvious, and most patients are diagnosed at late stage.^3^, ^4^ Currently, the endoscopic or surgical resection combined with chemotherapy is the only treatment option for GC patients. But the prognosis is very poor, with the average 5‐year survival rate being less than 20%.^4^ Conventional surgical resection is not a perfect treatment for advanced GC, and, multidrug resistance in refractory GC has become the most difficult obstacles to the success of chemotherapy and radiotherapy.^5^, ^6^ Hence, developing new drugs for eradicating GC is particularly urgent.

Demethylzeylasteral was originally regarded as a drug that has immunosuppressive and anti‐inflammatory effects.^7^ In recent years, some articles have confirmed that Dem has a significant inhibitory effect on various kinds of cancer, such as melanoma, glioma, and colorectal cancer.^8^, ^9^, ^10^ In addition, recent evidence has shown that Dem enhances the anti‐tumor activity of 5‐Fu in colorectal cancer cells, and it also elevates the chemotherapy sensitivity of pancreatic cancer cells to gemcitabine therapy.^10^, ^11^ However, the effects and underlying mechanisms of Dem on GC and its usage with 5‐Fu or DOX are unexplored.

The proto‐oncogene *c‐Myc* encodes the transcription factor c‐Myc, which has been reported to bind to 15% of the promoters of whole human genome.^12^ The c‐Myc expression and activity are strictly regulated due to its crucial roles in diverse cellular processes, such as cell differentiation, cell cycle progression, migration, invasion, and apoptosis involved in tumorigenesis and development.^13^, ^14^, ^15^, ^16^ c‐Myc is often overexpressed or over‐activated in many cancers, which is associated with the occurrence and development of various tumors.^13^ In most human tumor, its high expression and multiple downstream oncogenic pathways are often correlated with poor prognosis. Therefore, the regulation of c‐Myc stability is extremely an important issue. FBXW7, an E3 ubiquitin ligase, is a recognized tumor suppressor that mediates the degradation of various proteins including c‐Myc, CyclinE, and MCL‐1, whereby playing pivotal parts in regulating cell cycle, DNA damage and repair, signal transduction and transcription factors in tumor cells, respectively.^4^, ^12^, ^17^ Many studies have shown that FBXW7 expression is reversely correlated with enhanced tumor proliferation and migration.^18^, ^19^, ^20^ It has also been reported that decreased FBXW7 expression leads to upregulation of c‐Myc expression and is associated with a poor prognosis in cancer patients.^21^

In our study, we demonstrate that Dem induces GC cell apoptosis and also inhibits GC cell proliferation, migration, and invasion. We uncover that the downregulated c‐Myc expression via activation of FBXW7 contributes to these phenomenon and that Dem enhances the chemosensitivity of GC cells to 5‐Fu and DOX in vitro. Therefore, Dem may serve as a promising new anti‐cancer drug for GC treatment alone or in combination with other chemotherapeutics.

## 2 RESULTS

### 2.1 Demethylzeylasteral inhibits proliferation of GC cells

**FIGURE 1:**
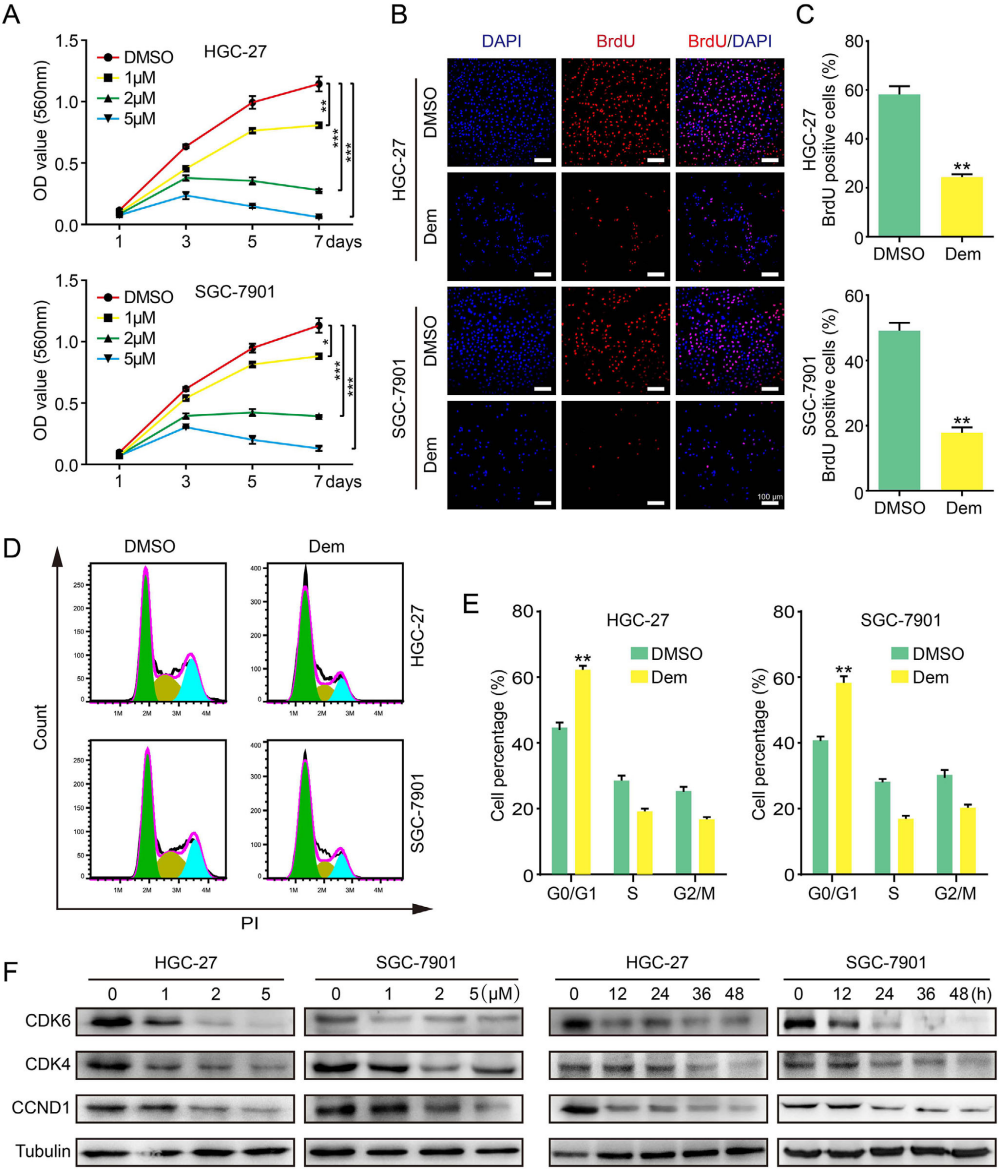
Demethylzeylasteral suppresses growth of GC cells. (A) The viability of GC cells was measured by MTT assay. Data were analyzed by three independent experiments ± SD. (B and C) Images and quantification of BrdU‐positive GC cells after treated with 2 µM Dem for 48 h, scale bar = 100µm. (D) Cell cycle was investigated via flow cytometry after 2 µM Dem treatment for 48 h. (E) The distribution ratio of G0/G1, S, and G2/M of panel D was determined. (F) The expression of cell cycle‐related proteins was detected by Western blot after different concentration Dem or 2µM Dem treatment for different time. DMSO was used as a control. ***p < 0.001, **p < 0.01, *p < 0.05

To study the effect of Dem on proliferation of GC cell, we used concentration gradient of Dem (1, 2, and 5 µM, DMSO as a control) to treat GC cells for 48 h, and the cell growth and proliferation were detected by MTT and BrdU assays. MTT assay manifested that, compared with DMSO group, the growth curve of GC cells treated with different concentrations of Dem decreased significantly, and their semi‐lethal concentrations (IC50) were approximately 4.1 µM and 5.3 µM for HGC‐27 and SGC‐7901 cells, respectively (Figures S1A and 1A). Microscopic observation revealed that the morphology of both cell lines changed significantly after Dem treatment, and the number of cells decreased with dose gradient effect (Figures S1B and S1C). BrdU test showed that compared with DMSO group, DNA synthesis of GC cells treated with 2 µM Dem for 48 h was significantly decreased (Figures 1B and 1C). Cells were farther analyzed by flow cytometry, and the data indicated that compared with DMSO group, the percentage of G0/G1 phase cells of GC was rose significantly after Dem treatment (Figures 1D and 1E), indicating that cell cycle was blocked at G0/G1 phase. To confirm these results, we checked the G0/G1‐related proteins CDK4, CDK6, and CCND1. The results showed that when treated with different concentrations (1, 2, and 5 µM; DMSO was utilized as a control) of Dem for 48 h or treated with 2 µM Dem for time gradient (0, 12, 24, 36, and 48 h), the expression of CDK4, CDK6, and CCND1 decreased in a dose and time gradient manner (Figure 1F).

### 2.2 Demethylzeylasteral induces apoptosis in GC cells

Many compounds inhibit tumor cell proliferation by inducing apoptosis. Therefore, we decided to test whether Dem causes GC cells apoptosis. The flow cytometry analysis showed that Dem could induce apoptosis in GC cells after treated with 5 µM for 48 h (Figures S2A and S2B). To farther verify the results, we treated the cells with different concentrations (2, 5, and 10 µM; DMSO was utilized as a control) of Dem for 48 h or 5 µM Dem for time gradient (0, 12, 24, 36 and 48 h). The apoptosis‐related proteins, including cleaved‐caspase3 (C‐Caspase3), cleaved‐PARP (C‐PARP), PARP, and anti‐apoptotic protein BCL2 showed a dose‐dependent and time‐dependent change (Figures S2C and S2D). The above results indicated that Dem induces GC cell apoptosis.

### 2.3 Demethylzeylasteral inhibits migration and invasion of GC cells

**FIGURE 2:**
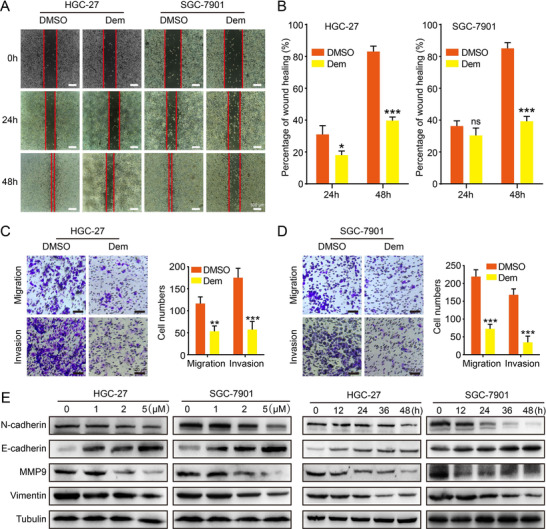
Demethylzeylasteral inhibits migration and invasion of GC cells. (A and B) The wound healing ability and quantification of GC cells after 2 µM Dem treatment, scale bar = 500 µm. (C and D) Pictures and quantification of the ability of migration and invasion about GC cells after treatment with 2 µM Dem, scale bar = 100 µm. (E) The expression of metastasis‐related proteins was detected by Western blot after different concentration Dem treatment for 48 h or 2 µM Dem treatment for different time. DMSO was used as a control. ***p < 0.001, **p < 0.01, *p < 0.05

In view of the high metastasis and rapid proliferation of GC cells, we evaluated the impacts of Dem treatment on the ability of migration and invasion by transwell and wound healing assay. The results of wound healing assay suggested that Dem dramatically prolonged the time of wound‐healing (Figures 2A and S2B). In addition, the transwell assay farther showed that the ability of migration and invasion in GC cells after Dem treatment were significantly inhibited (Figures 2C and 2D). Lastly, Western blot (WB) assay was used to test the expression level of N‐cadherin, E‐cadherin, MMP9 and Vimentin, which play a crucial part in cell migration and invasion. The expression of N‐cadherin, MMP9, Vimentin was down‐regulated, and in contrast, the expression of E‐cadherin was upregulated in cells treated with Dem (Figure 2E). In conclusion, these results indicate that Dem inhibits human GC cells migration and invasion.

### 2.4 Overexpression of c‐Myc rescues demethylzeylasteral‐inhibited GC cells proliferation, migration, and invasion

**FIGURE 3:**
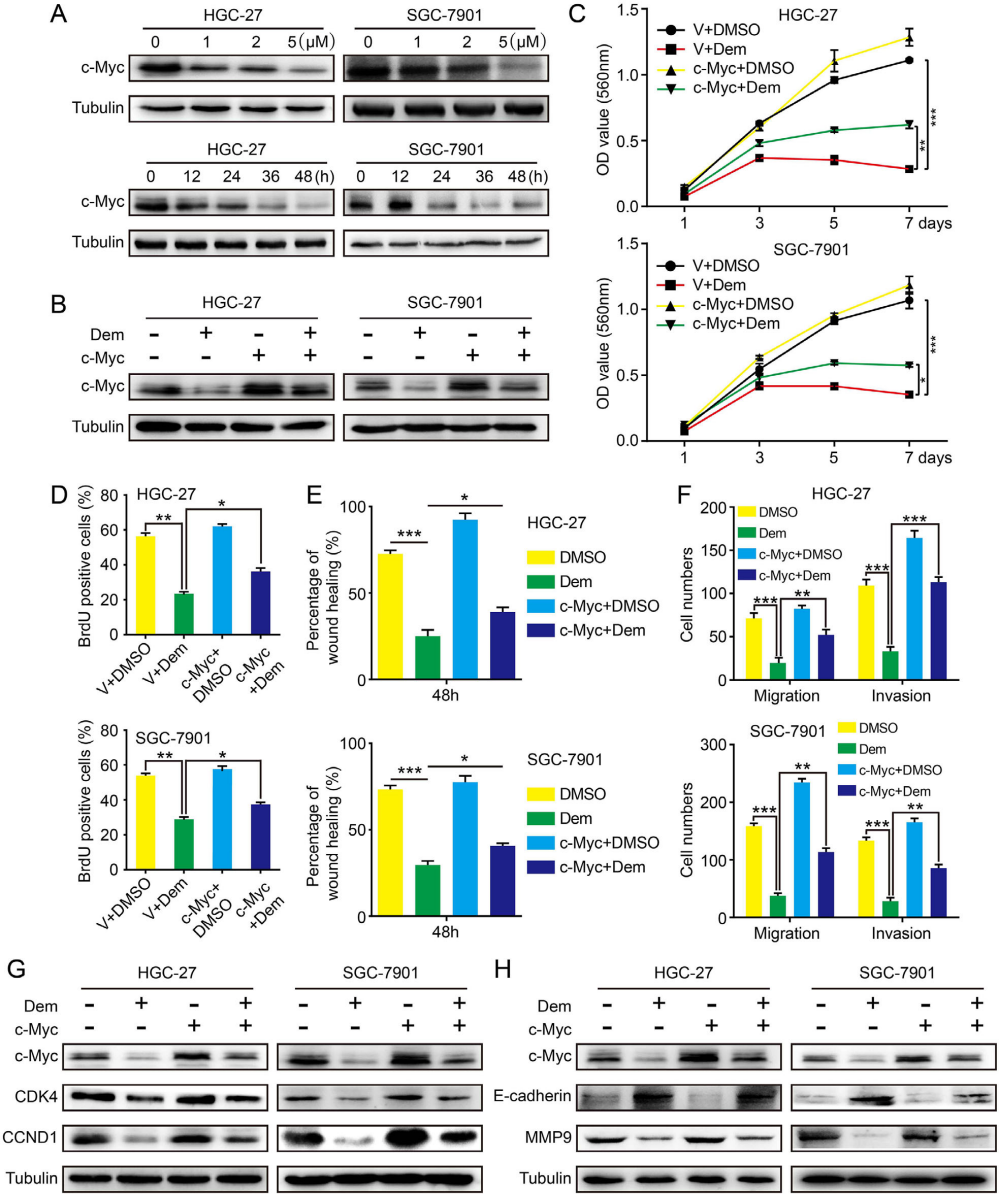
c‐Myc overexpression restores GC cell proliferation, migration, and invasion inhibited by Demethylzeylasteral. (A) The expression of c‐Myc was detected by Western blot after different concentration Dem treatment for 48 h or 2 µM Dem treatment for different time. (B) The c‐Myc expression of cells overexpressing c‐Myc or empty vector was detected after 2 µM Dem treatment. (C) Growth curve of GC cells overexpressing c‐Myc or empty vector after 2 µM Dem treatment. The data were analyzed by three independent experiments ± SD. (D) Quantification of BrdU‐positive GC cells overexpressing c‐Myc after treated with 2 µM Dem. (E) The effect of wound closure in GC cells overexpressing c‐Myc after treated with 2 µM Dem. (F) The quantification of migration and invasion of GC cells overexpressing c‐Myc after treated with Dem. (G) CDK4 and CCND1 expressions of c‐Myc‐overexpressed GC cells were tested by Western blot after 2 µM Dem treatment. (H) E‐cadherin and MMP9 expression of c‐Myc overexpressed GC cells were tested by Western blot after 2 µM Dem treatment. ***p < 0.001, **p < 0.01, *p < 0.05

We employed the Protein Data Bank (PDB) and IGEMDOCK to validate the Dem‐target binding capacity. Results showed that Dem had great binding activity to c‐Myc (Figure S3A). We therefore checked the oncoprotein c‐Myc's expression, which plays a vital part in cell cycle and cell proliferation. The results indicated that c‐Myc was downregulated in a dose and time‐dependent manner after Dem treatment (Figure 3A). The lentivirus encoding c‐Myc was used to infect GC cells, then WB showed that c‐Myc expression was upregulated compared with empty vector and DMSO group after infection (Figure 3B). MTT assay also described that compared with empty vector groups, the cells growth rate of c‐Myc‐overexpressing treated with Dem was significantly higher (Figure 3C). BrdU staining depicted that c‐Myc overexpression rescued the DNA synthesis that was reduced by Dem (Figures 3D and S3B). Wound healing assay showed that c‐Myc‐overexpressing cells dramatically decreased the time of wound‐healing compared with the group of empty vector treated with Dem (Figures 3E and S3C). In addition, the transwell assay revealed that c‐Myc overexpression could partially restore cell migration and invasion ability compared with the vector group treated with Dem (Figures 3F and S3D). Subsequently, WB results further confirmed that the related cyclins (CDK4 and CCND1) were partially recovered after c‐Myc overexpression under Dem treatment (Figure 3G). Besides, E‐cadherin and MMP9 were partially restored in c‐Myc overexpression group with Dem treatment (Figure 3H). In summary, our results indicate that c‐Myc partly rescues GC cell proliferation, migration, and invasion, which are inhibited by Dem.

### 2.5 Demethylzeylasteral decreases the c‐Myc protein stability

**FIGURE 4:**
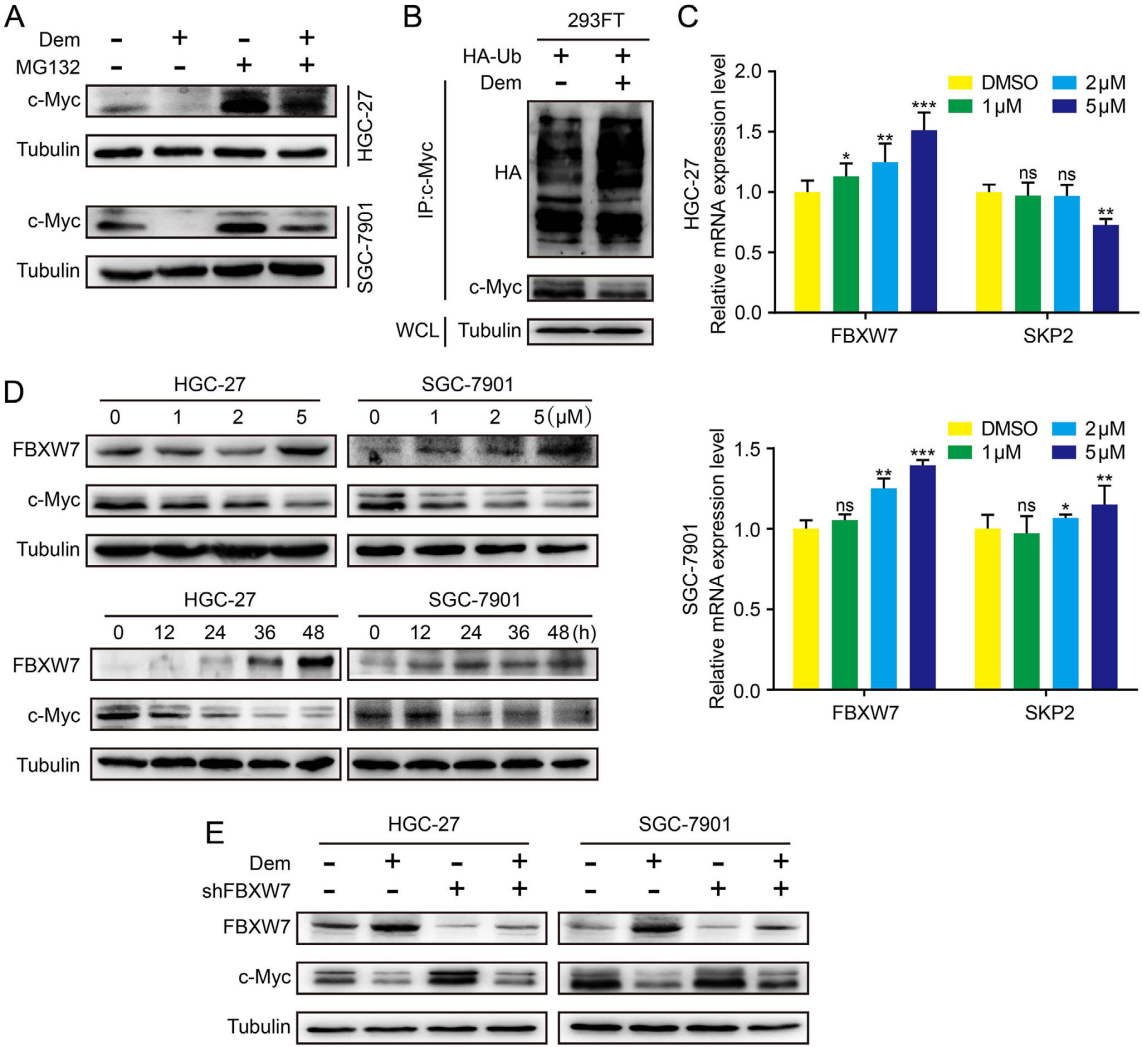
Demethylzeylasteral decreases the stability of c‐Myc protein by FBXW7. (A) The effect of Dem and MG132 on c‐Myc expression in GC cells was tested by Western blot. (B) The ubiquitination of c‐Myc in 293‐FT cells was enhanced after Dem treatment. (C) The mRNA level of FBXW7 and SKP2 in GC cells after Dem treatment was detected by qPCR. (D) The expression of FBXW7 and c‐Myc in GC cells after Dem treatment was detected by Western blot. (E) The effects of FBXW7 knockdown on protein expression in Dem treated cells were analyzed by Western blot. ***p < 0.001, **p < 0.01, *p < 0.05

In order to further study the underlying molecular mechanism of Dem‐regulated c‐Myc, quantitative real‐time (qRT)‐PCR experiment was performed. The results indicated that c‐Myc mRNA level was not decreased after Dem treatment (Figure S4A). Therefore, we conjectured that Dem might impact the c‐Myc protein stability. Exactly, WB analysis showed that MG132 could partially save the reduction of c‐Myc in GC cells treated with Dem (Figure 4A). Further, we found that Dem upregulates the ubiquitylation levels of c‐Myc (Figure 4B). Next, the related proteins that regulate the ubiquitylation level of c‐Myc were analyzed through qRT‐PCR. The results suggested that the expression of E3 ubiquitin ligase FBXW7 was upregulated after Dem treatment (Figures 4C and S4B). WB also presented that the FBXW7 protein level was upregulated in a dose‐dependent and time‐dependent manner (Figure 4D). When FBXW7 was downregulated in GC cells, Dem‐induced expression c‐Myc partially restored (Figure 4E). Therefore, Dem inhibits c‐Myc expression through activating the FBXW7‐mediated ubiquitylation, whereby inhibiting GC cell proliferation, migration, and invasion.

### 2.6 Demethylzeylasteral inhibits self‐renewal and tumorigenesis in GC cells through down‐regulating c‐Myc expression

**FIGURE 5:**
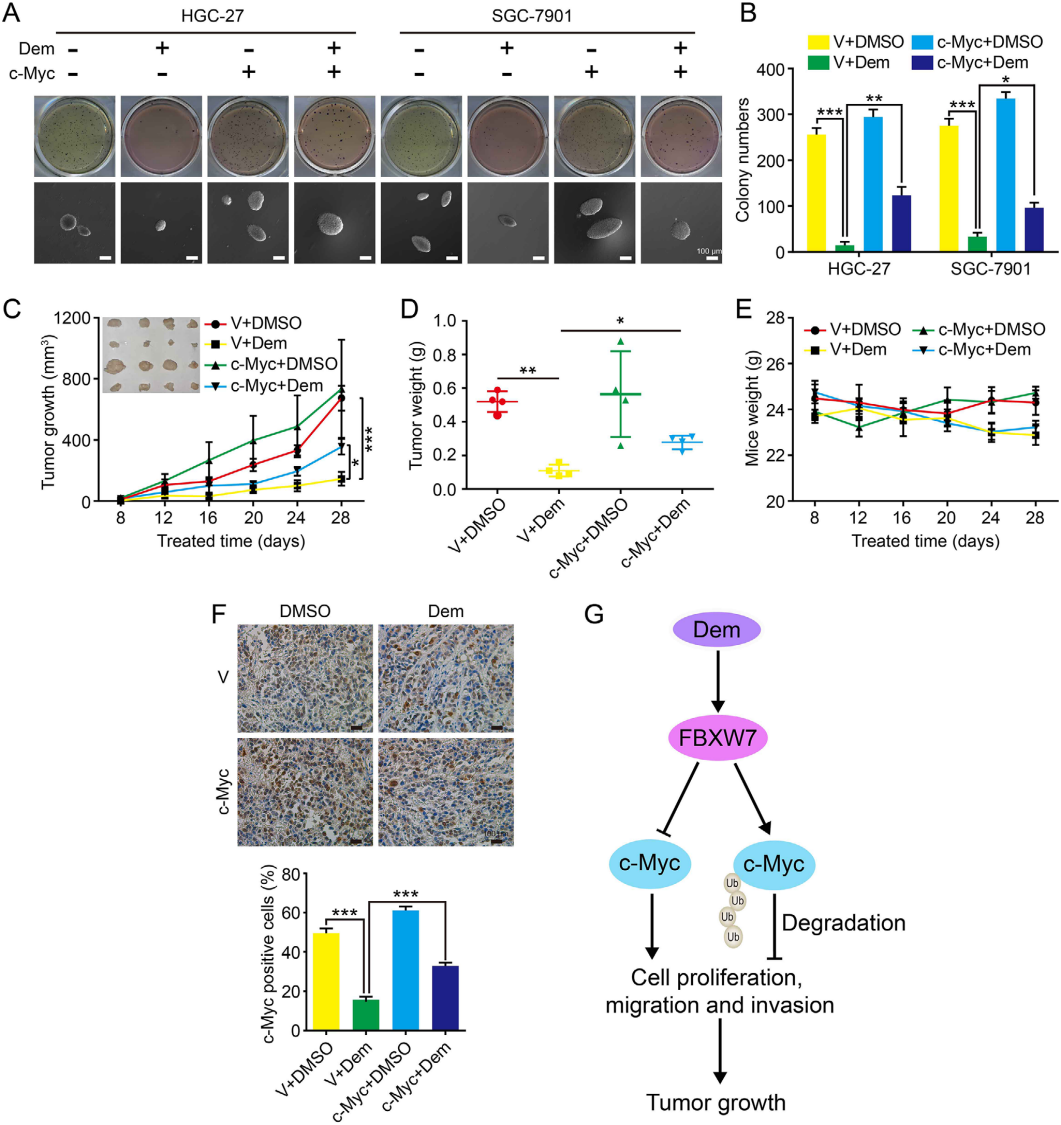
Demethylzeylasteral suppresses clonogenicity and tumor growth of GC through down‐regulating the expression of c‐Myc. (A) Soft agar assay was performed with c‐Myc or empty vector overexpressed GC cells after DMSO or Dem treatment, scale bar = 100 µm. (B) The numbers of clones in panel (A) was counted. (C) Tumor volume and photograph of tumors from indicated mice treated with DMSO or Dem were shown. (D) Tumor weights of indicated mice treated with DMSO or Dem were shown. (E) The weights of mice in each group were measured. (F) c‐Myc overexpression in indicated tumors was investigated by IHC, scale bar = 100 µm. (G) Summary of the mechanism by which Dem inhibits cell proliferation and tumor progression in human GC. ***p < 0.001, **p < 0.01, *p < 0.05

In order to study whether Dem has an effect on the self‐renewal ability of GC cells, soft agar assay was performed. Results indicated that compared with DMSO group, the group treated with 2 µM Dem had smaller and fewer colonies, but overexpression of c‐Myc could rescue this phenomenon (Figures 5A and 5B). Meanwhile, HGC‐27 cells were subcutaneously transplanted into NOD/SCID mice. Consequences showed that compared with the control group injected with DMSO, lower tumor weight and smaller tumor size were found in the mice injected with Dem. Furthermore, injecting HGC‐27 cells overexpressing c‐Myc could rescue this phenomenon (Figures 5C and 5D). There is no significant weight change in mice treated with Dem or with tumors overexpressing c‐Myc, compared with control group (Figure 5E). IHC staining with c‐Myc also showed that c‐Myc overexpression rescued the suppressive effect of Dem on GC (Figure 5F). In summary, these data indicated that Dem suppressed tumor growth through regulating the FBXW7/c‐Myc axis in GC (Figure 5G).

### 2.7 Demethylzeylasteral enhances chemosensitivity of GC cells to doxorubicin and 5‐Fluorouracil in vitro

**FIGURE 6:**
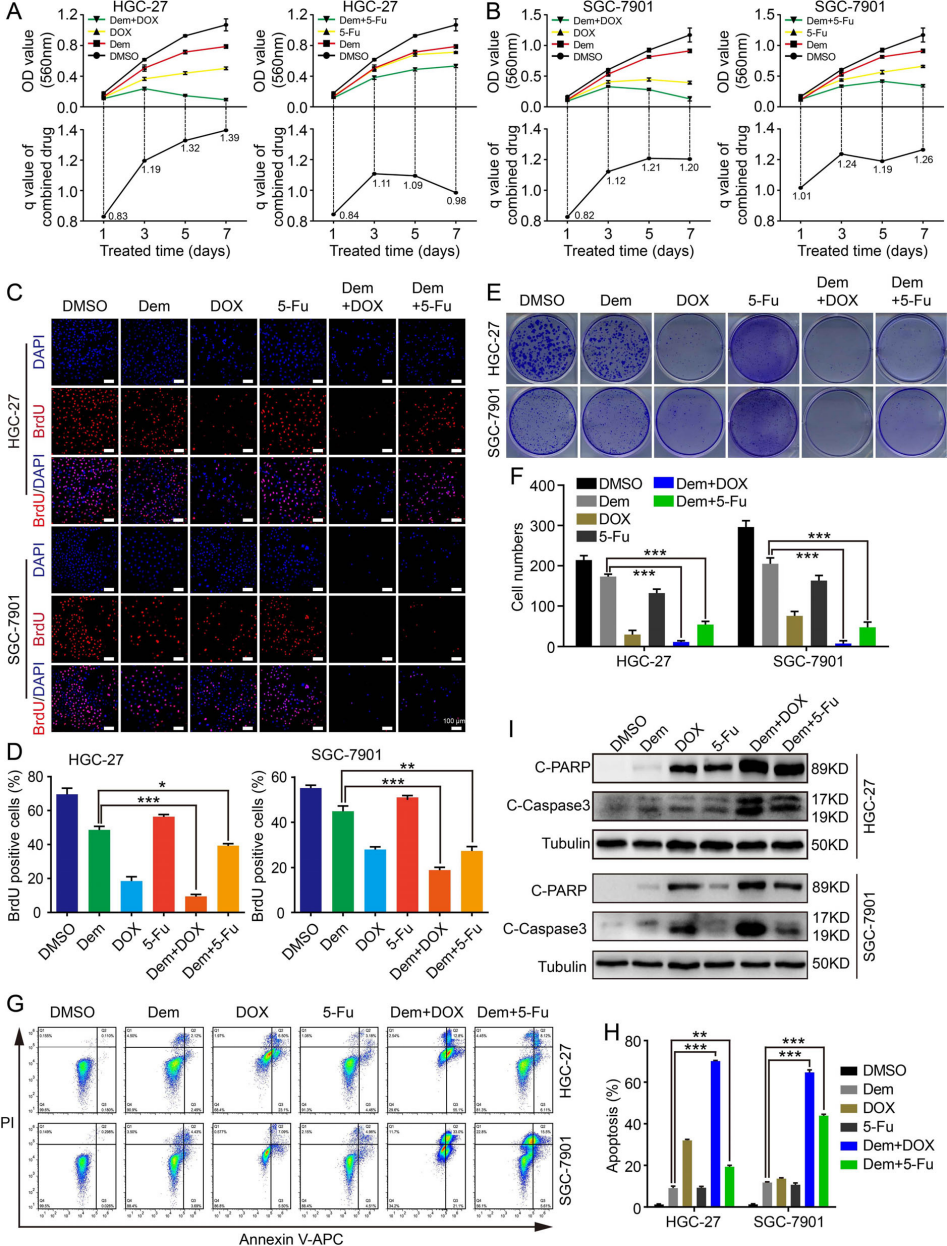
Demethylzeylasteral enhances GC cell chemosensitivity to Doxorubicin and 5‐Fluorouracil in vitro. (A and B) Growth curve of GC cells with different treatment (1 µM Dem, 0.1 µM DOX, 150 µM 5‐Fu, 1 µM Dem+0.1 µM DOX, 1 µM Dem+150 µM 5‐Fu) was shown. The efficiency index (q) analysis of Dem combined with 5‐Fu or DOX treatment was determined through Jin's formula. (C) BrdU staining assay was performed in different drug combination groups, scale bar = 100 µm. (D) BrdU‐positive rates in panel (C) were quantified. (E) Clone formation assay was performed in different drug combination groups. (F) Clone numbers in panel (E) were quantified. (G) Apoptosis in different drug combination groups was detected. (H) Apoptotic rates in panel (G) were quantified. (I) The apoptotic proteins expression of were detected by Western blot. ***p < 0.001, **p < 0.01, *p < 0.05

At last, we tested the synergistic effect of Dem with DOX and 5‐Fu in GC cells, and two commonly used anti‐cancer drugs for GC. The MTT assay showed that DOX or 5‐FU combined with Dem had stronger inhibitory effects compared with single drugs through Jin's formula (Figures 6A and 6B). The synergistic effect of Dem+5‐Fu in HGC‐27 cells was not satisfactory. BrdU staining experiment revealed that the BrdU‐positive rate of DOX or 5‐Fu combined with Dem was lower than that of the single drug (Figures 6C and 6D). Meanwhile, clone formation assay also proved that DOX or 5‐Fu combined with Dem inhibited cell proliferation more severe than treated alone (Figures 6E and 6F). Moreover, DOX or 5‐Fu combined with Dem resulted in a higher rate of apoptosis in GC cells than the single drug (Figures 6G and 6H). The expression levels of apoptosis‐related proteins C‐PARP and C‐Caspase3 after the combination drugs were significantly higher than that of single drug revealed by WB assay (Figure 6I). These data suggest that Dem enhances the chemosensitivity to DOX or 5‐Fu by inducing apoptosis of GC cells in vitro.

## 3 DISCUSSION

Dem is a monomer extracted from *Tripterygium wilfordii* Hook F. Since its discovery, there have been articles reporting that Dem has immunosuppressive and anti‐inflammatory effects.^7^, ^22^ However, over the past few years, many researchers have shown that it can effectively inhibit tumor development. At present, it has been reported that Dem has anti‐tumor effect in melanoma, glioma, colorectal cancer, and so on.^8^, ^9^, ^10^, ^11^, ^23^ The latest study found that Dem could induce apoptosis in GC MKN‐45 cell line.^24^ However, its specific anti‐tumor mechanism in GC still remains vague. Meanwhile, our research found that Dem can not only induce apoptosis in GC cell lines‐HGC‐27 and SGC‐7901 (Figures S2A‐S2D), but also inhibit cell proliferation, migration, and invasion by regulating FBXW7/c‐Myc axis. Our study shows that Dem may have the potential for clinical treatment in the future.

The transcriptional activator c‐Myc controls a variety of transcriptional programs and takes a pivotal part in the development of many human cancers. The expression of c‐Myc is strictly controlled by multiple mechanisms in the cell under normal physiological conditions.^25^ Although normal cell growth and proliferation really require the c‐Myc, the deregulation of c‐Myc activation or overexpression is associated with the occurrence and development of most human cancers.^26^, ^27^ The c‐Myc protein is overexpressed in many human cancers.^28^, ^29^ It has been proved that its direct inhibition triggers fast tumor degeneration in mice with only slight and completely invertible side effects, indicating that this may be a feasible treatment strategy.^30^, ^31^, ^32^ c‐Myc protein, as a short half‐life protein, is strictly controlled by the ubiquitin proteasome system. At present, more than 10 ubiquitin ligases have been uncovered to ubiquitinate c‐Myc, and many deubiquitinases can inhibit this process, such as ubiquitin ligase FBXW7, SKP2 and deubiquitin ligase USP28, USP36 and so on.^12^, ^33^, ^34^, ^35^, ^36^ We further verified that Dem did not downregulate c‐Myc mRNA level. However, the level of c‐Myc protein was downregulated. Therefore, we conjectured that Dem could impact the c‐Myc stability through direct binding, indirect regulation or the above two manners. Thereafter, qRT‐PCR assays were performed on FBXW7, SKP2, USP28, and USP36, which confirmed that Dem could interact with FBXW7 and downregulate the expression of c‐Myc. Downregulation of FBXW7 could significantly inhibit the downregulation of c‐Myc induced by Dem. Therefore, we proved that Dem may inhibit the proliferation, migration, and invasion of GC cells mainly via the FBXW7/c‐Myc axis. However, the direct binding of Dem with c‐Myc still needs to be further verified.

GC is one of the most pervasive cancers in China.^37^ While chemotherapy has made encouraging progress so far in treating tumors, the benefits of chemotherapeutic drug‐based treatment are often impaired by the development of chemotherapy resistance.^38^, ^39^ Hence, it is crucial to enhance the sensitivity of chemotherapy drugs. 5‐Fu and DOX are commonly used clinical anti‐tumor chemotherapy drugs, which have inhibitory effects on a variety of human tumors, mainly by inducing tumor cell apoptosis.^40^, ^41^, ^42^, ^43^, ^44^, ^45^, ^46^ The present study showed that Dem can enhance the chemotherapy sensitivity of pancreatic cancer cells to gemcitabine and colorectal cancer cells to 5‐Fu.^10^, ^11^ But it is unclear whether Dem can enhance the chemotherapy sensitivity of 5‐Fu or DOX to GC cells. Therefore, we explored the effect of Dem on GC cells when combined with 5‐Fu or DOX. Compared with the single drug, when Dem was used in combination with 5‐Fu or DOX, the inhibitory effect on GC cells was more obvious, and it could significantly induce cell apoptosis (Figure 6). Therefore, it may provide a more promising combination therapy strategy for cancer treatment. Further research will focus on investigating the molecular mechanisms of this synergistic effect.

## 4 MATERIALS AND METHODS

### 4.1 Reagents and antibodies

The antibodies were bought from Cell Signaling Technology (c‐Myc, CDK4, CDK6, CCND1, BCL2, C‐PARP, PARP, and C‐Caspase 3), Proteintech Group (N‐cadherin, E‐cadherin, MMP9, Vimentin, FBXW7, α‐Tubulin, and HA‐tag), Bioss (c‐Myc for IHC), Abcam (anti‐BrdU), and Beyotime (HRP goat‐rabbit and goat anti‐mouse). Phenylmethylsulfonyl Fluoride (PMSF), RIPA lysis buffer, and BCA protein assay kit were bought from Beyotime. 5‐Fu and DOX were obtained from Med Chem Express and Selleck Chemicals, respectively. 5‐Bromo‐2‐deoxyuridine (BrdU), 3‐(4, 5‐dimethylthiazol‐2‐yl)‐2, 5‐diphenyltetrazolium bromide (MTT), dimethyl sulfoxide (DMSO), and MG132 were purchased from Sigma‐Aldrich. 4’, ‐6‐Diamidino‐2‐phenylindole (DAPI) and puromycin were obtained from Life Technologies. The Hieff Trans Liposomal Transfection Reagent was purchased from Yeasen. ECL solution was offered by US Everbright Inc.

### 4.2 Cell culture

RPMI1640 (Biological Industries [BI]) medium with 10% fetal bovine serum (BI) and 1% penicillin‐streptomycin (BI) was used to culture GC (HGC‐27 and SGC‐7901) cells. DMEM (BI) medium with 2% L‐glutamine (BI), 1% G418 (BI), 1% sodium pyruvate (BI), and 1% non‐essential amino acids (BI) was used to culture 293FT cells.

### 4.3 Drug treatment

Demethylzeylasteral (Dem, C_29_H_36_O_6_) was obtained from Must (Chengdu, China) and dissolved in DMSO as the stock solution with concentration at 10 mM. Different concentrations of Dem (1, 2, 5 µM) were used to treat GC cells for dose‐dependent experimental, and 2 µM Dem was used for different time treatment (0, 12, 24, 36, and 48 h) for time‐dependent experiment. DMSO was used as negative control.

### 4.4 MTT assay

To detect the cell viability, HGC‐27and SGC‐7901 cells were seeded in 96‐well plate as 1000 cells per well (200 µL medium/well). Afterwards cells were cultured with different concentrations of Dem (0, 1, 2, 5 µM) for several days. MTT was added into each well (5 mg/ml, 20 µL/well) for 2 h incubation. Then microplate reader was used to measure the absorbance at 560 nm after formazan was dissolved in 150µL DMSO.

### 4.5 BrdU staining

The cell's proliferative ability was measured by BrdU staining accordingly. The detailed procedures were mentioned previously.^8^

### 4.6 Flow cytometry analysis

Flow cytometry was used for analysis of apoptosis and cell cycle. The detailed steps were as before.^8^

### 4.7 WB assay

Cells were lysed with RIPA Lysis Buffer with PMSF, and protein concentrations were measured by BCA protein assay kit. Then the proteins were used for SDS‐PAGE. After that the proteins were transferred to polyvinylidene difluoride membrane. Five percent skim milk in TBST was used to block the membrane at RT for 2 h, after that the corresponding diluted primary antibody was used to incubate the membrane at 4°C overnight. And then the secondary antibody was used for 1.5 h RT incubation. The results finally were visualized by the ECL Prime WB analysis though the ECL reagent.

### 4.8 qRT‐PCR (Quantitative real‐time polymerase chain reaction)

RNAiso (TaKaRa) was used for total RNA extraction according to the manufacture's instruction. Then, 2 µg RNA was used to produce cDNA by reverse transcription for each sample. LightCycle 96 real‐time PCR system (Roche) was utilized for qRT‐PCR. GAPDH was used as reference gene. Primers used were listed as follows:

MYC‐F: GGCTCCTGGCAAAAGGTCA;
MYC‐R: CTGCGTAGTTGTGCTGATGT;
FBXW7‐F: GGCCAAAATGATTCCCAGCAA;
FBXW7‐R: ACTGGAGTTCGTGACACTGTTA;
Skp2‐F: ATGCCCCAATCTTGTCCATCT;
Skp2‐R: CACCGACTGAGTGATAGGTGT;
USP28‐F: CACTGTTGCTACAGAACCATCT;
USP28‐R: TGGGAGACTCCAGTAGACTCA;
USP36‐F: TCTGCCAAGAAGGTCCTTTTACA;
USP36‐R: TGGCGACTAGCTCCCTCTG;
GAPDH‐F: CACGGATTTGGTCGTATTGGGC;
GAPDH‐R: CTGATTTTGGAGGGATCTCGCC.

### 4.9 Wound healing, migration, and invasion assay

2 µM Dem was used for all these experiments with DMSO served as control. The specific experimental method was described as before.^47^

### 4.10 Transfection and infection

The c‐Myc‐overexpression plasmid was obtained from YouBio, and the empty vector plasmid was offered by Addgene. The shFBXW7 and shGFP (negative control) plasmid were obtained from Sigma‐Aidrich. Transfection and infection follow the steps below: firstly, every single targeting plasmid (c‐Myc‐overexpression, GFP‐overexpression, shGFP, or shFBXW7), and three packaging plasmids including pLP/VSVG, pLP1, pLP2 were equally co‐transfected into 293FT cells using the liposomes when the density of 293FT cells was about 90%. Secondly, the virus was harvested 48 h later, and then GC cells were infected with virus containing polybrene. Finally, the cells were screened with puromycin and passaged.

### 4.11 Soft agar and colony formation assays

GC cells’ self‐renewal and colony formation ability were detected by soft agar and colony formation assays. For the soft agar assay, 1.5 ml 2 × RPMI‐1640 (Gibco) including 0.6% agarose (Sigma) on each well of six‐well plate and then replenish 1 ml complete medium containing 800 cells, 0.3% agar, and 2 µM Dem after below layer solidified. After cultured in 37°C incubator about 2 weeks, colonies were captured by microscopy and scanned after MTT stained. For colony formation assay, seed 800 cells each well of six‐well plate and add different drugs (Dem, DOX, 5‐Fu, Dem+DOX, Dem+5‐Fu) for about 2 weeks treatment after the cells adhered. Stain cells with 0.1% crystal violet and then scan.

### 4.12 Subcutaneous tumor xenografts

Four‐week‐old Non‐Obese Diabetic/Severe Combined Immunodeficiency Disease (NOD/SCID) mice were purchased and divided into two groups and kept in SPF room. 1 × 10^6^ cells overexpression c‐Myc or GFP were suspended with 100 µl medium and then injected to flanks of each group mice, respectively. About 1 or 2 weeks later, divide each group into two more groups, one is injected with Dem (5 mg/kg) and the other with the same amount of DMSO. Each group was injected every other day for 2 weeks. Mice weight and tumor volume were measured every 4 days. (tumor volume = (π/6) × length × width^2^).^8^

### 4.13 Ubiquitination assay

293FT cells were treated with HA‐Ub plasmid and then treated with 2 µM Dem for 48 h. Add MG132 (50 µg/ml) into the medium 8 h before harvested. c‐Myc antibody was used for Immunoprecipitation and HA‐tag antibody for WB to detect ubiquitination level. See details in previous study.^4^

### 4.14 Immunohistochemical (IHC) assay

After immobilization and dehydration, xenograft tumors were paraffin embedded and sectioned. c‐Myc antibody was used for overnight incubation of tissue slice at 4°C, and replaced by secondary antibody for 20 min incubation at RT. Afterwards, stain the sections with DAB and hematoxylin, and capture photographs.

### 4.15 Jin's formula

We assessed the drug‐combined effect in vitro through Jin's formula.^4^, ^48^ The Jin's formula is as follows:

$$\begin{array}{c} q=\frac{E(A+B)}{\left(\mathrm{EA}+\mathrm{EB}-\mathrm{EA}*\mathrm{EB}\right)}\\ \begin{array}{cc} \text{Annotate:} & \\ q:\;\text{Efficiency}\;\text{index} & q<0.85:\;\text{antagonism}\;\text{effect}\\ E(A+B):\;\text{Combined}\;\text{inhibition}\;\text{rate} & 0.85\;\leq \;q\;<\;1.15:\;\text{superosition}\;\text{effect}\\ \text{EA:}\;\text{Drug}\;A\;\text{inhibition}\;\text{rate} & q\geq 1.15:\;\text{synergistic}\;\text{effect}\\ \mathrm{EB}:\;\text{Drug}\;B\;\text{inhibition}\;\text{rate} & \end{array} \end{array}$$

### 4.16 Molecular docking

Structure of the c‐Myc was downloaded from Protein Data Bank (RCSB PDB, website: https://www.rcsb.org/). Molecular docking analysis of Dem with c‐Myc was performed by IGEMDOCK tool. Bond energy score was identified to judge the degree of intermolecular bonding.

### 4.17 Statistical analysis

The Graphpad prism software was used to analyze statistical data. Data were shown as MEAN ± SD (standard deviation) and analyzed by unpaired two‐tailed *t*‐test. *p < 0.05, **p < 0.01, and ***p < 0.001 were considered statistically significant (ns: no significant).

## CONFLICT OF INTEREST

The authors have no conflict of interest.

## ETHICS APPROVAL

All animal experiments were approved by the animal ethics committee of Southwest University and performed humanly in accordance with guidelines of the Care and Use of Laboratory Animals (Ministry of Science and Technology of China, 2006).

## AVAILABILITY OF DATA AND MATERIALS

All the data and materials in this manuscript are freely and publicly available.

## AUTHOR CONTRIBUTIONS

Yongsen Li, Yongyue Su, Hongjuan Cui, and Muhan Lü designed the experiments. Yongsen Li, Yuzu Zhao, Xiaosong Hu, and Gaichao Zhao performed the experiment. Jiang He and Sicheng Wan analyzed the data and prepared figures. Yongsen Li wrote the manuscript. Yongyue Su, Hongjuan Cui, and Muhan Lü revised the manuscript.

## Supporting information

Supporting information

Supporting information

Supporting information

Supporting information

Supporting information
