# Erratum

**DOI:** 10.1002/mco2.142

**Published:** 2022-05-02

On the first publication of MCO273, the authors noted that images of SGC‐7901 group in Figure 1F were misplaced by mistake. The corrected Figure 1F is now shown in this correction. The authors confirm that the conclusions of this paper are not affected, and sincerely apologize for this error and any inconvenience that may have caused.

**FIGURE 1:**
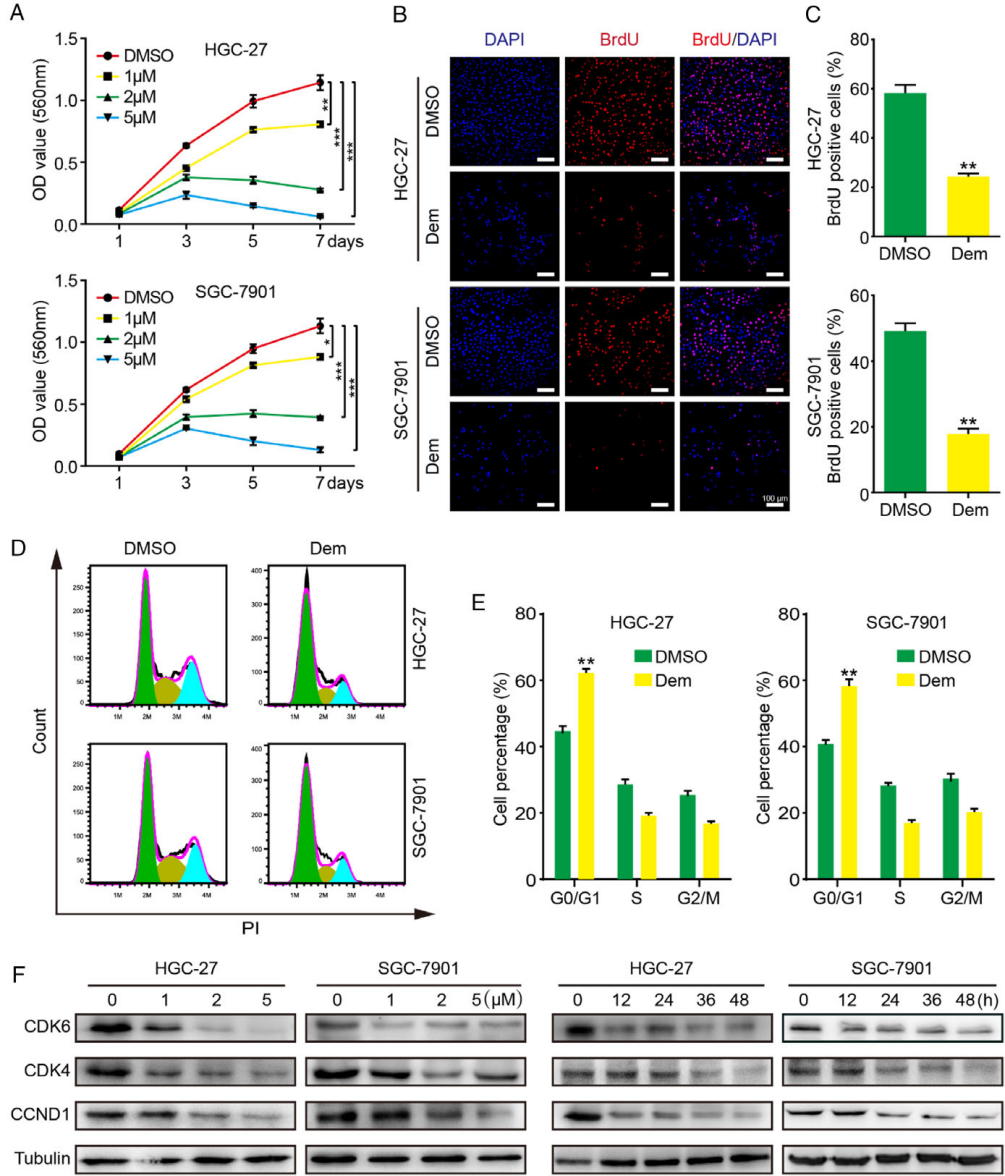
Demethylzeylasteral suppresses growth of GC cells. (A) The viability of GC cells was measured by MTT assay. Data wereanalyzed by three independent experiments ± SD. (B and C) Images and quantification of BrdU‐positive GC cells after treated with 2 μM Demfor 48 h, scale bar = 100μm. (D) Cell cycle was investigated via flow cytometry after 2 μM Dem treatment for 48 h. (E) The distribution ratio ofG0/G1, S, and G2/M of panel D was determined. (F) The expression of cell cycle‐related proteins was detected by Western blot after differentconcentration Dem or 2 μM Dem treatment for different time. DMSO was used as a control. *** *p* <0.001, ** *p* <0.01, * *p* <0.05
