# Supplementary material for: Demethylzeylasteral inhibits proliferation, migration, and invasion through FBXW7/c‐Myc axis in gastric cancer
Source: MedComm (2020). 2021 Jun 3;2(3):467–80. doi: 10.1002/mco2.73 (PMC8554662; doi:10.1002/mco2.73)
Supplement: Supplementary file 1 — Supporting information [file MCO2-2-467-s004.docx]

**Supplementary Information**

**
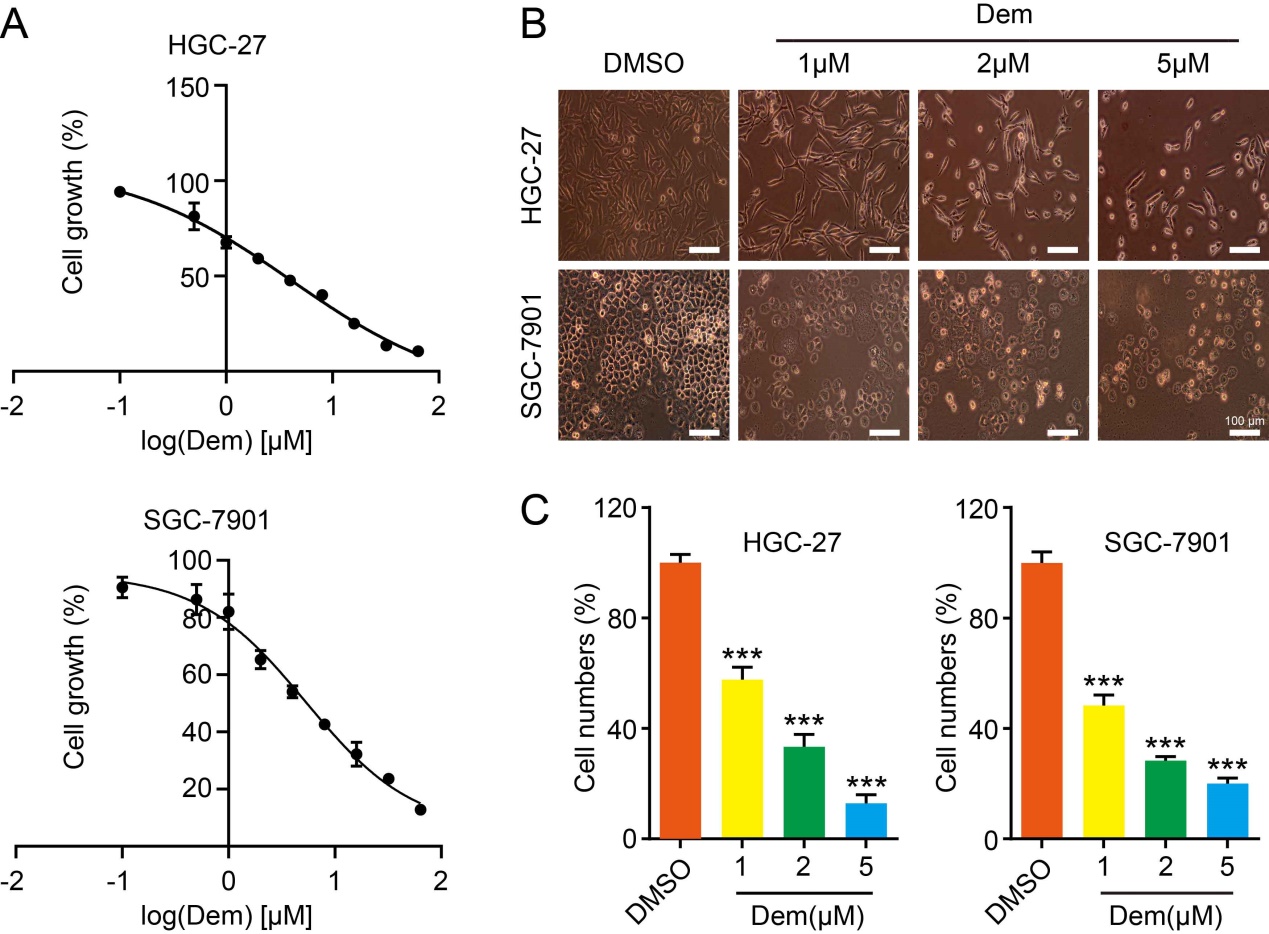
**

**Figure S1 Demethylzeylasteral inhibits proliferation of GC cells. A** The viability of GC cells after treatment with 0.1, 0.5, 1, 2, 4, 8, 16, 32 and 64 μM Dem. **B** The morphology of GC cells treated with gradients concentration of Dem for 48 h, scale bar=100μm. **C** The numbers of GC cells were counted and the DMSO treated group was regard as 100%. ****p*<0.001, ***p*<0.01, **p*<0.05


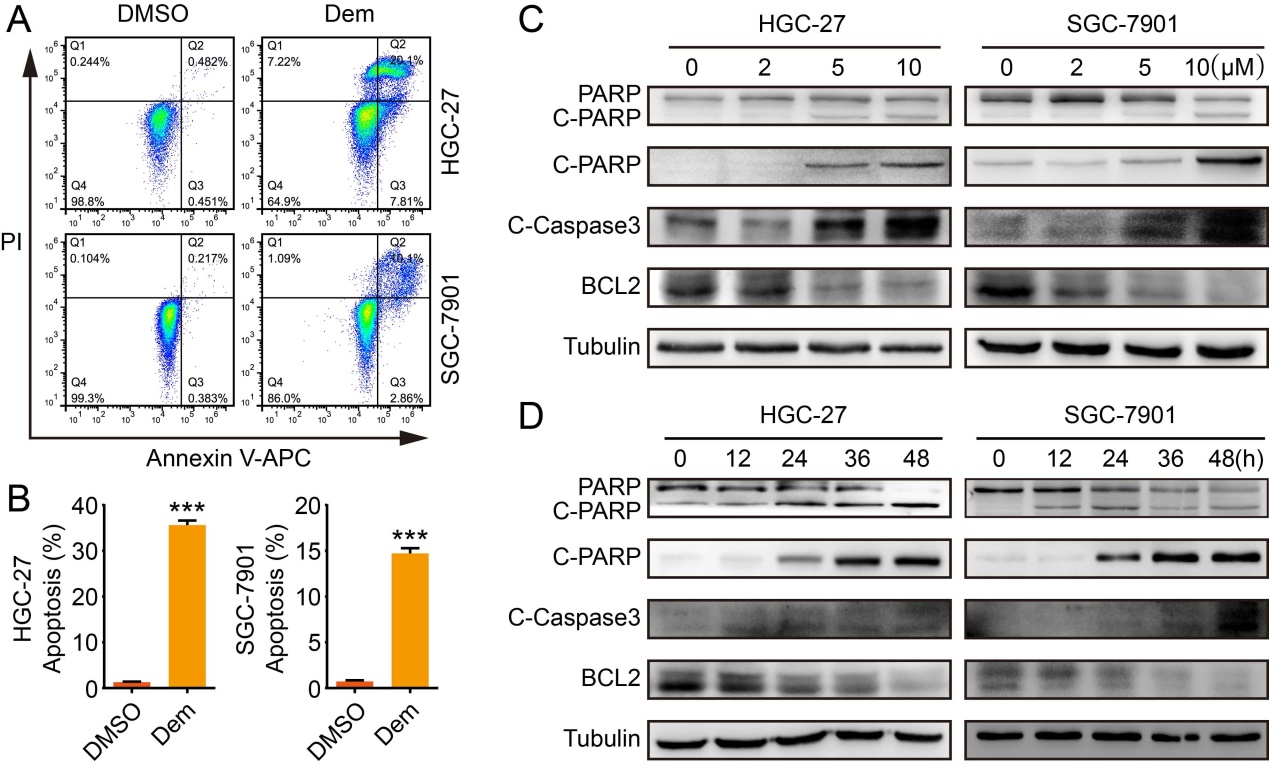


**Figure S2 Demethylzeylasteral induces apoptosis in GC cells. A, B** Apoptosis rate of GC cells treated with 5 μM Dem for 48 h were examined by flow cytometry. **C, D** The apoptotic proteins expression was detected by Western blot. ****p*<0.001, ***p*<0.01, **p*<0.05


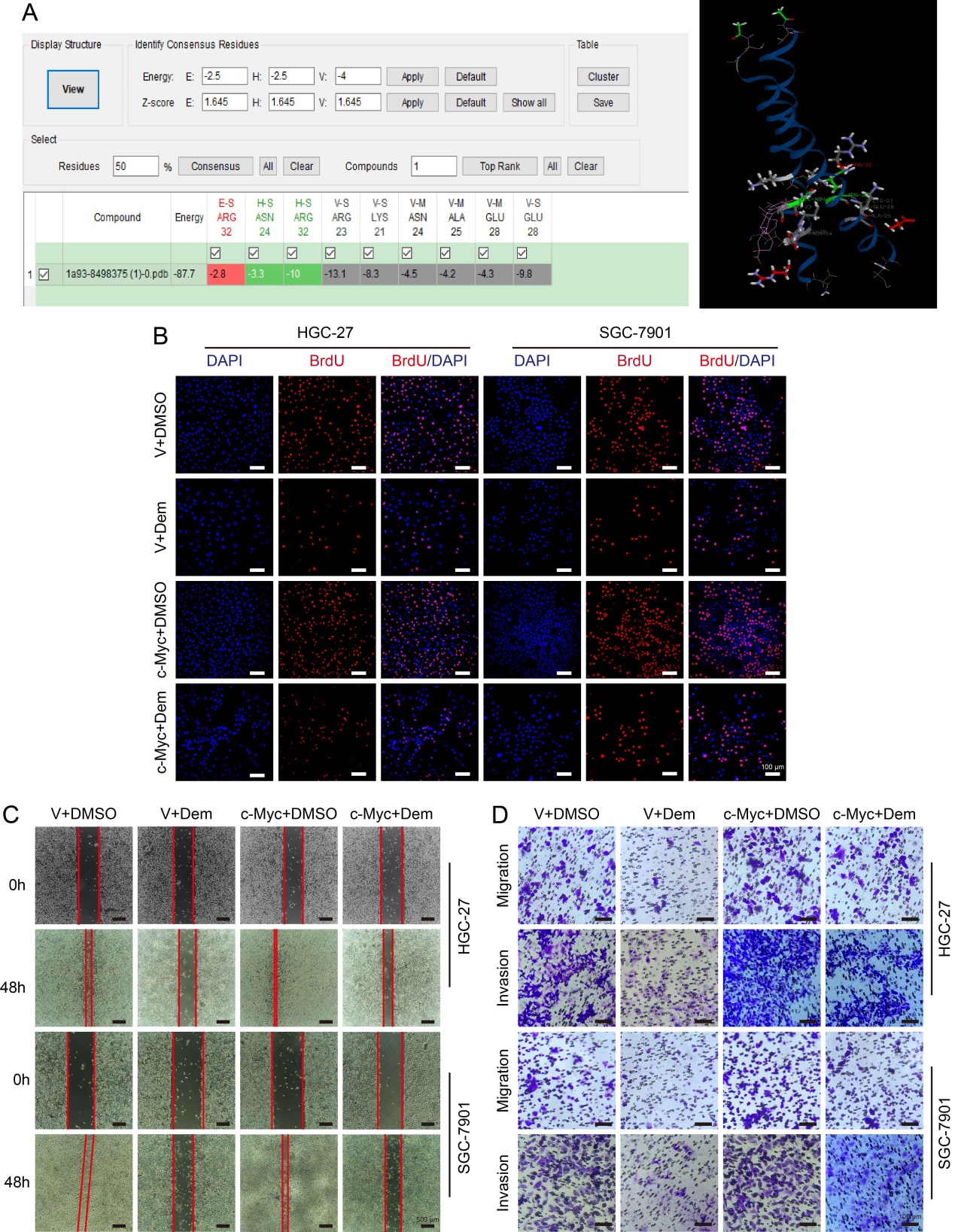


**Figure S3 Demethylzeylasteral inhibits GC cells migration and invasion. A** The interactions and 3D virtual docking structure of Dem and c-Myc. **B** BrdU staining assay was performed overexpressing c-Myc after treated with 2 μM Dem, scale bar=100μm. **C** Wound closure of GC cells overexpressing c-Myc after treated with 2 μM Dem, scale bar=500μm. **D** Migration and invasion of GC cells overexpressing c-Myc after treated with 2 μM Dem, scale bar=100μm. ****p*<0.001, ***p*<0.01, **p*<0.05


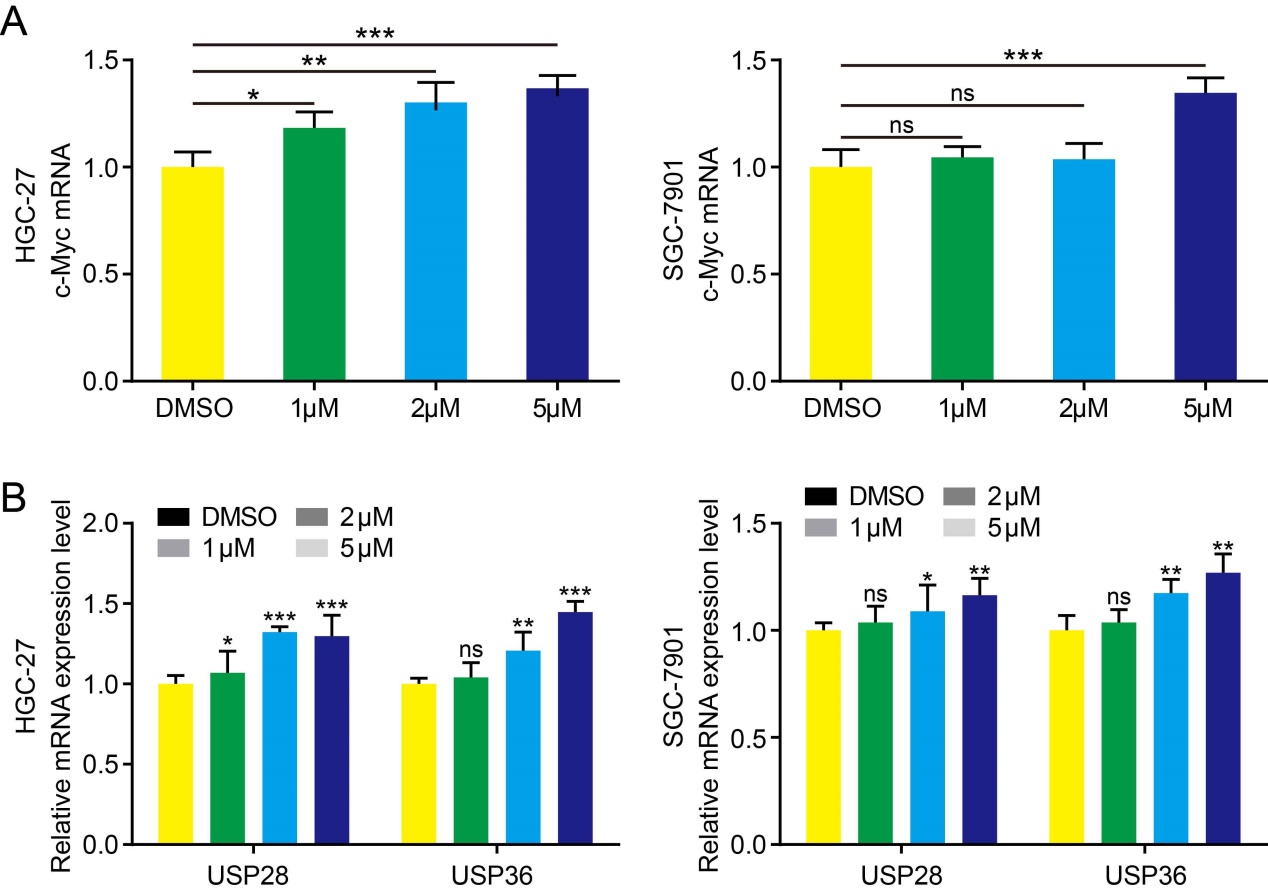


**Figure S4 Demethylzeylasteral decreases the stability of c-Myc protein through FBXW7. A** qPCR was performed to detect the mRNA level of c-Myc in GC cells after Dem treatment. **B** qPCR was performed to detect the mRNA level of USP28 and USP36 in GC cells after Dem treatment. ****p*<0.001, ***p*<0.01, **p*<0.05
