# Supplementary figures and images for: Demethylzeylasteral inhibits proliferation, migration, and invasion through FBXW7/c‐Myc axis in gastric cancer
Source: MedComm (2020). 2021 Jun 3;2(3):467–80. doi: 10.1002/mco2.73 (PMC8554662; doi:10.1002/mco2.73)

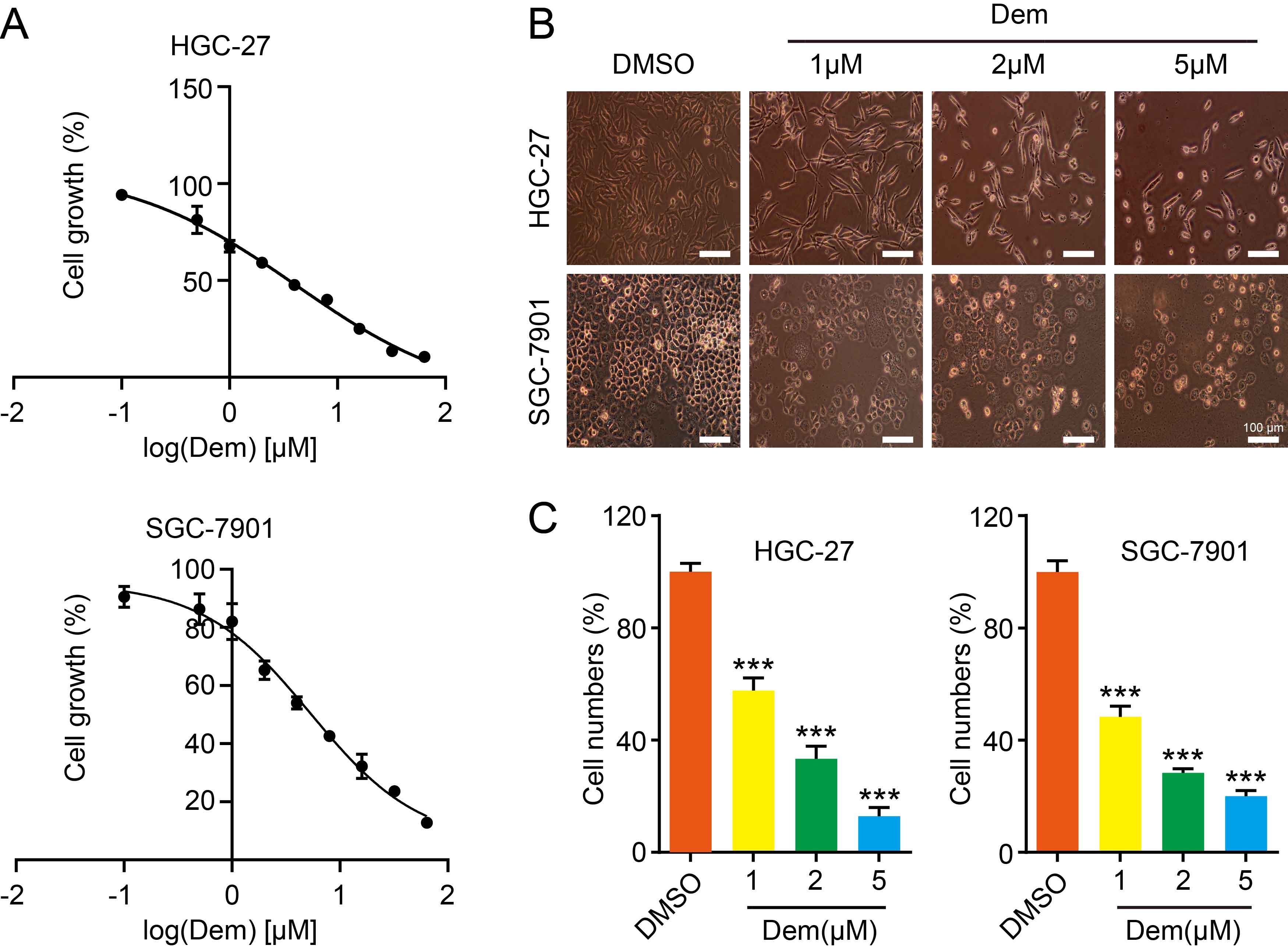

Supplement: Supplementary file 2 — Supporting information [file MCO2-2-467-s003.jpg]

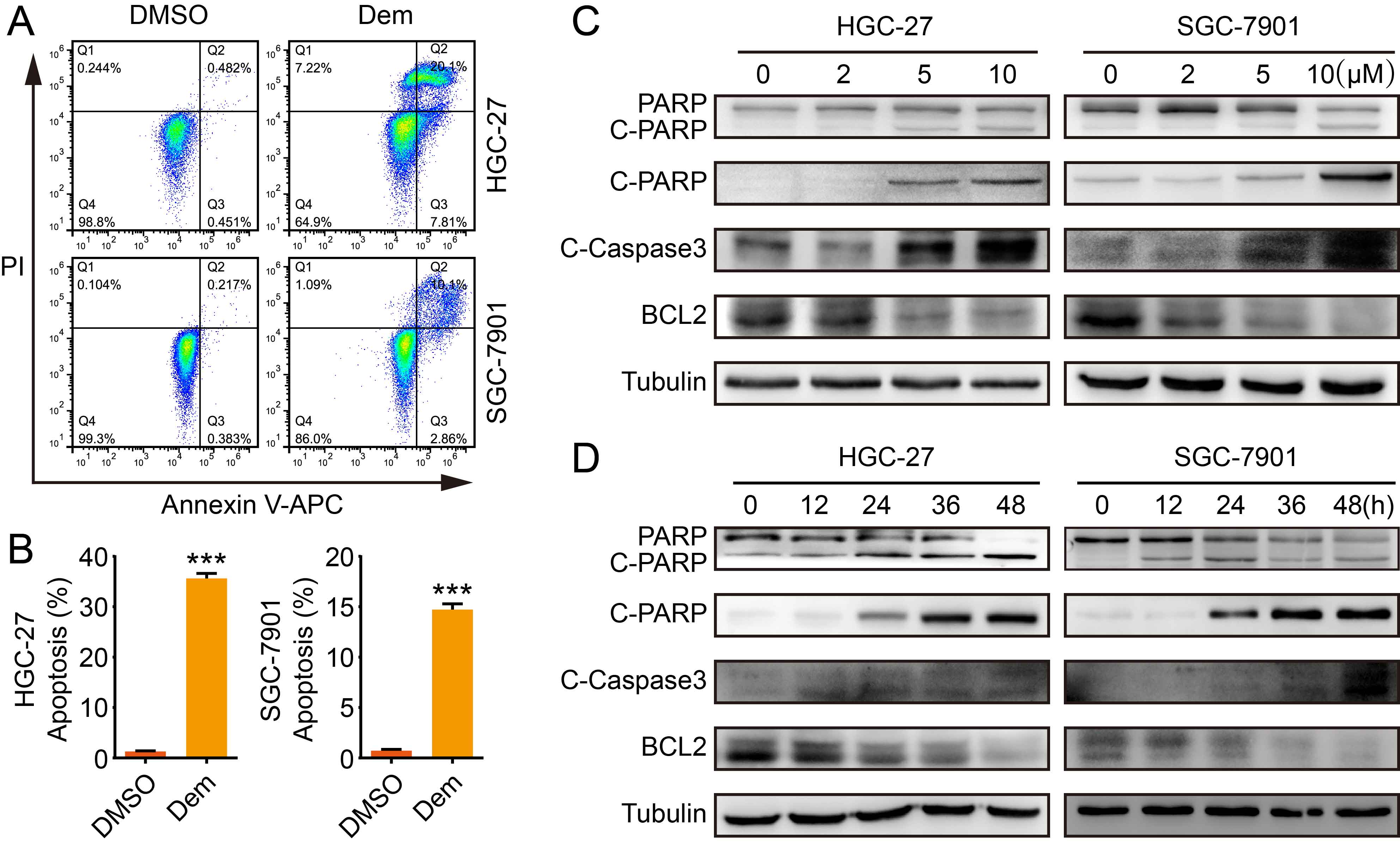

Supplement: Supplementary file 3 — Supporting information [file MCO2-2-467-s002.jpg]

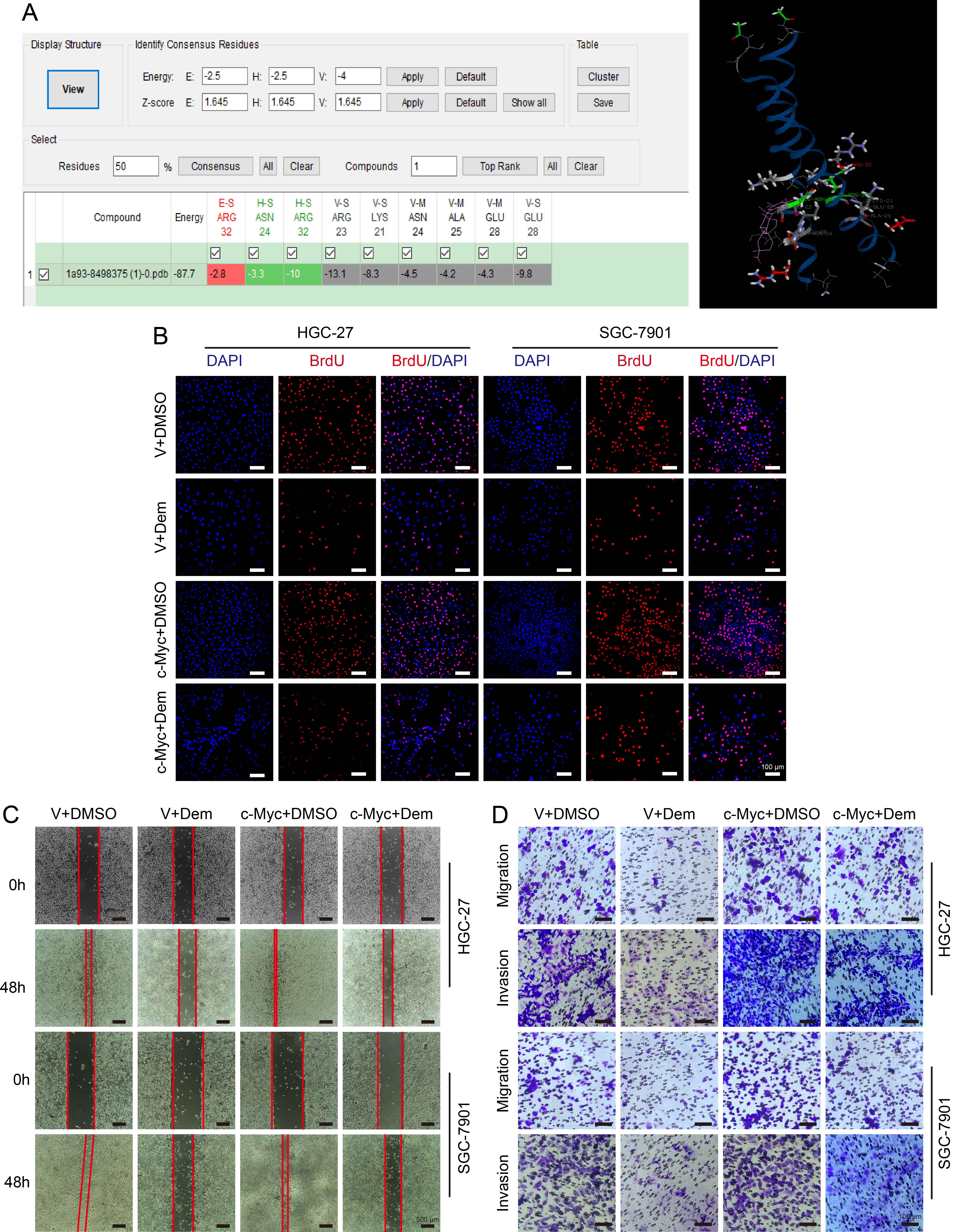

Supplement: Supplementary file 4 — Supporting information [file MCO2-2-467-s005.jpg]

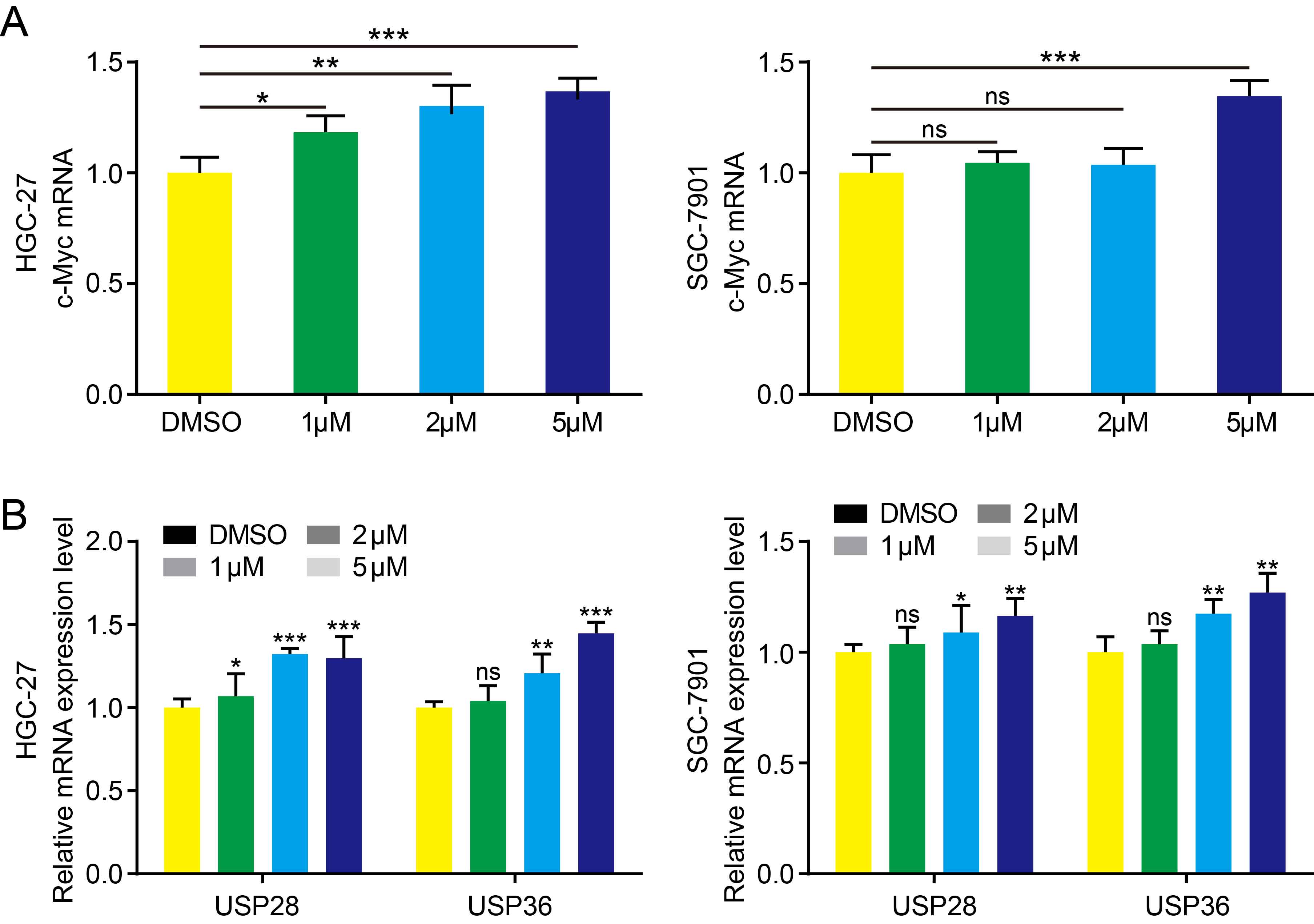

Supplement: Supplementary file 5 — Supporting information [file MCO2-2-467-s001.jpg]
